# Supplementary material for: Photochromic and Luminescent Properties of a Salt of a Hybrid Molecule Based on C60 Fullerene and Spiropyran—A Promising Approach to the Creation of Anticancer Drugs
Source: Molecules. 2023 Jan 22;28(3):1107. doi: 10.3390/molecules28031107 (PMC9921579; doi:10.3390/molecules28031107)
Supplement: Supplementary file 1 [file molecules-28-01107-s001.zip › molecules-2143739-supplementary.pdf]

Supplementary information

The ^{1}H and ^{13}C NMR spectra were run on a Bruker Avance-500 spectrometer at 500.17 and 125.78 MHz or on a Bruker Avance-400.13 spectrometer at 400.17 and 100.62 MHz, respectively. $CDCl_3$ was used as a solvent. High resolution mass spectrometry (HRMS) was performed on a MaXis impact, Bruker. Thin-layer chromatography was performed on silica gel plates (Sorbfil TLC, CTX-1VE) to monitor the reactions. Spots were made visible with UV light. Column chromatography was performed with silica gel (Sigma-Aldrich, high-purity grade, 60 Å/63–200 μm).

The spectrophotometric measurements were performed on a Cary 60 UV-Vis Spectrophotometer in 2- and 10-mm thick quartz cells. For photochemical measurements the solutions were irradiated by an L8253 xenon lamp included in an LC-8 radiation unit (Hamamatsu) at a medium radiation power through colour glass filters, UFS-1 and SZS-9.

The photoluminescence (PL) and excitation spectra, luminescence lifetimes of test compounds were registered using Horiba Jobin Yvon Fluorolog-3 spectrofluorometer (model FL-3-22) equipped with doublegrating monochromators, dual lamp housing containing a 450 W Xenon lamp, laser-diode NanoLED, photomultiplier tube detector (Hamamatsu R928 PMT) and InGaAs photodiode (DSS-IGA020 L) for IR measurements. For measurements of luminescence lifetimes were used a pulsed (frequency is 1 MHz) laser-diode NanoLED-370 (λ_{exc} = 369 nm), NanoLED-455 (λ_{exc} = 453 nm) and NanoLED-590 (λ_{exc} = 592 nm). The PL and excitation spectra were corrected in all cases for source intensity (lamp and grating) and emission spectral response (detector and grating) by standard instrument correction provided in the instrument software FluorEssence 3.5.

Compound 1: ^{1}H NMR (500 MHz, $CDCl_3$): δ = 1.19 (s, 3H), 1.30 (s, 3H), 2.03(m, 2H), 2.52 (s, 5H), 2.75 (t, *J* = 6.7 Hz, 2H), 3.35 (m, 1H), 3.43 (m, 1H), 5.93 (d, *J* = 10.3 Hz, 1H), 6.74 (t, *J* = 8.0 Hz, 2H), 6.99 (d, *J* = 10.3 Hz, 1H), 7.61 (s, 1H), 7.69 (d, *J* = 7.6, 1H), 8.00 (s, 2H), 9.78 (s, 1H) ppm. ^{13}C NMR (125 MHz, $CDCl_3$): δ = 19.81, 25.45, 25.93, 41.01, 44.03, 52.15, 56.20, 106.15, 106.19, 115.55, 118.25, 120.77, 122.09, 122.96, 126.11, 128.88, 129.52, 134.24, 136.99, 141.36, 152.09, 158.64, 190.50 ppm.

The desired spiropyran was synthesized using methods reported in the literature [1,2].

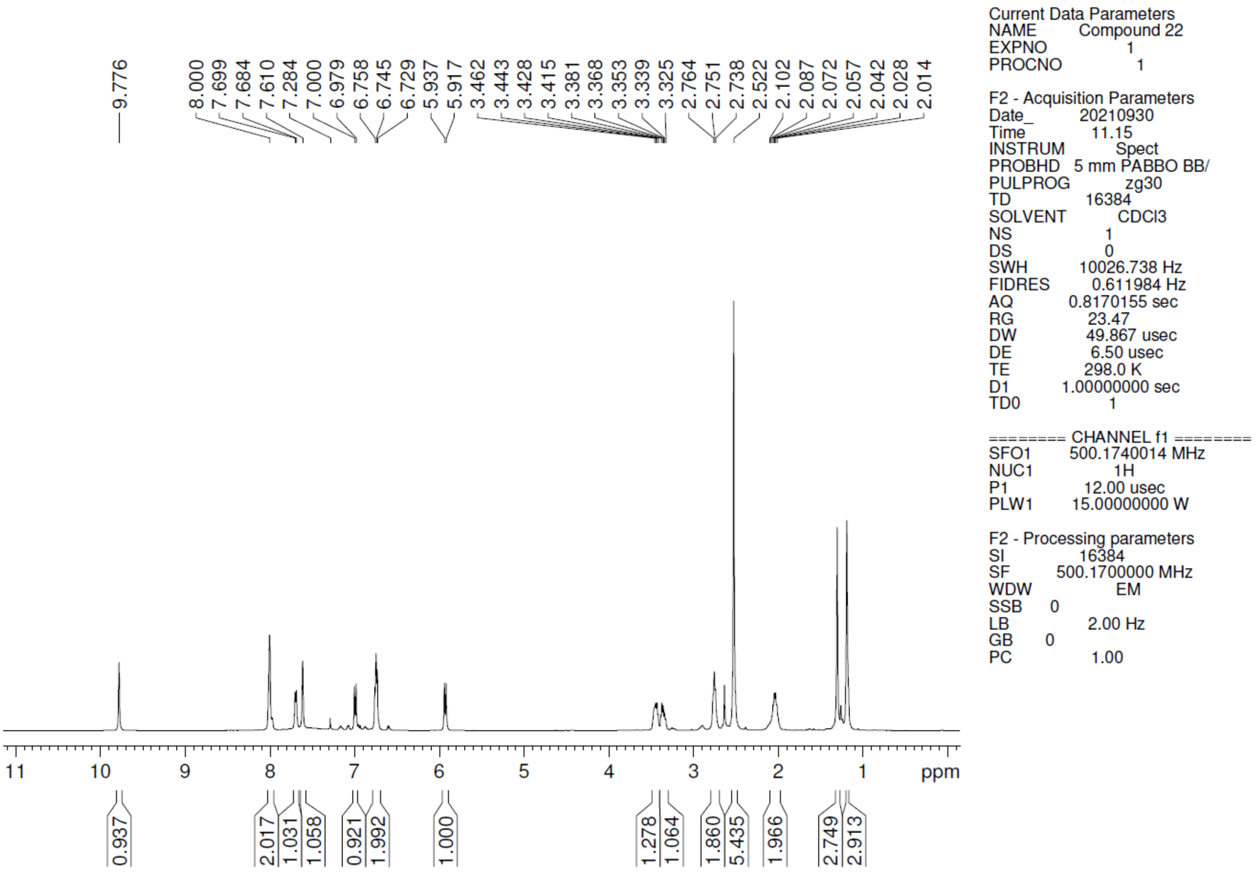

Figure S1. ^{1}H NMR spectra of compound **1** (500,17 MHz, $CDCl_3$).

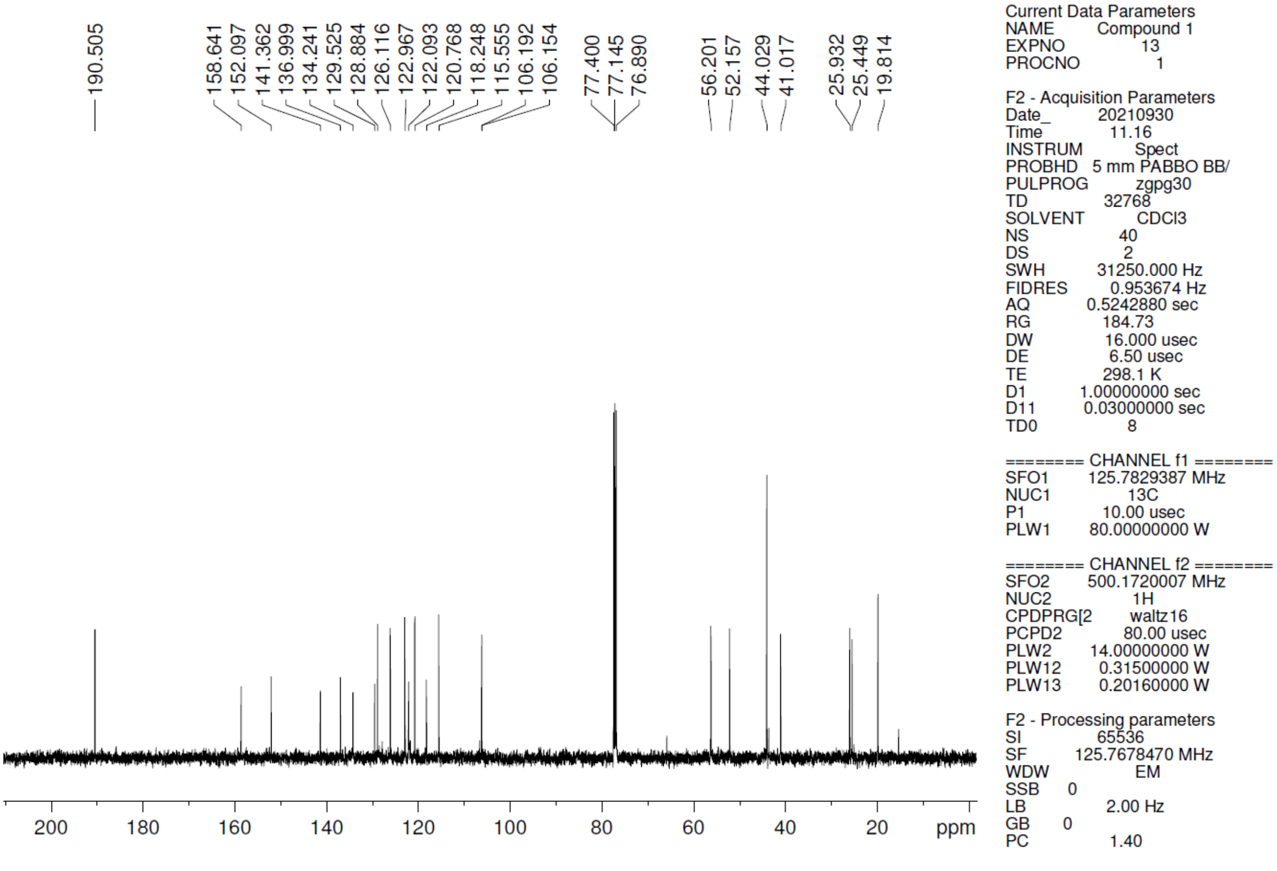

Figure S2. ^{13}C NMR spectra of compound **1** (125,78 MHz, $CDCl_3$).

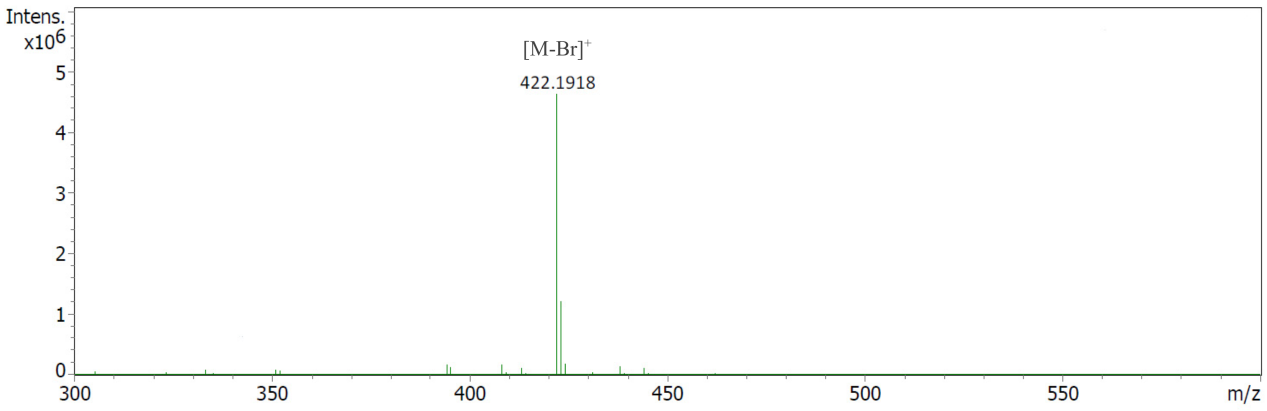

Figure S3. ESI-HRMS spectra of compound **1**.

Compound 2: ^{1}H NMR (500 MHz, $CDCl_3$): δ = 1.22 (br. s, 3H), 1.35 (br. s, 3H), 1.70 (m, 2H), 1.79 (m, 2H), 2.15 (s, 3H), 2.18 (s, 3H), 2.25 (m, 3H), 2.92 (s, 3H), 3.23 (m, 2H), 4.30 (d, J = 9.3 Hz, 1H), 4.94 (s, 1H), 5.01 (d, J = 9.3 Hz, 1H), 5.80 (d, J = 10.4 Hz, 1H), 6.60 (dd, J = 18.6, 7.2 Hz, 1H), 6.89 (dd, J = 10.3, 6.3 Hz, 1H), 7.99 (d, J = 12.7 Hz, 1H), 8.04 (d, J = 7.7 Hz, 1H) ppm. ^{13}C NMR (125 MHz, $CDCl_3$): δ = 19.87, 25.80, 27.08, 40.16, 41.68, 45.39, 52.70, 52.73, 57.27, 68.85, 70.06, 77.89, 77.95, 83.66, 106.71, 115.43, 115.52, 118.47, 121.85, 121.89, 122.70, 122.75, 125.90, 127.72, 127.77, 128.14, 135.88, 136.38, 136.54, 139.26, 139.33, 139.83, 140.10, 140.12, 140.20, 140.93, 140.97, 141.59, 141.70, 141.88, 141.94, 141.98, 142.01, 142.05, 142.14, 142.19, 142.28, 142.61, 142.72, 143.01, 143.12, 143.21, 144.39, 144.43, 144.66, 144.69, 144.72, 145.10, 145.13, 145.16, 145.26, 145.32, 145.40, 145.41, 145.45, 145.54, 145.58, 145.83, 145.86, 145.96, 146.13, 146.19, 146.24, 146.32, 146.36, 146.52, 146.58, 147.06, 147.31, 147.34, 153.68, 154.15, 154.30, 156.48, 159.52 ppm.

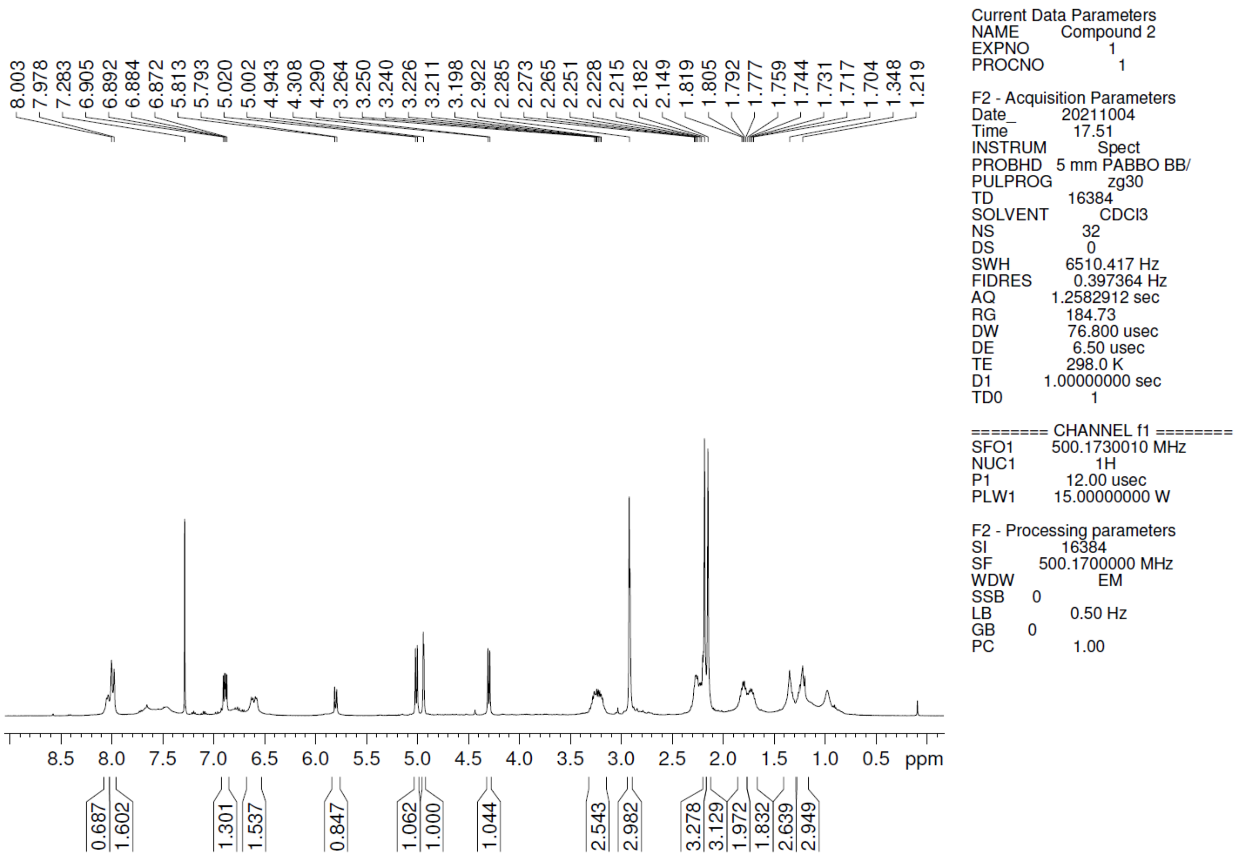

Figure S4. ^{1}H NMR spectra of compound **2** (500,17 MHz, $CDCl_3$).

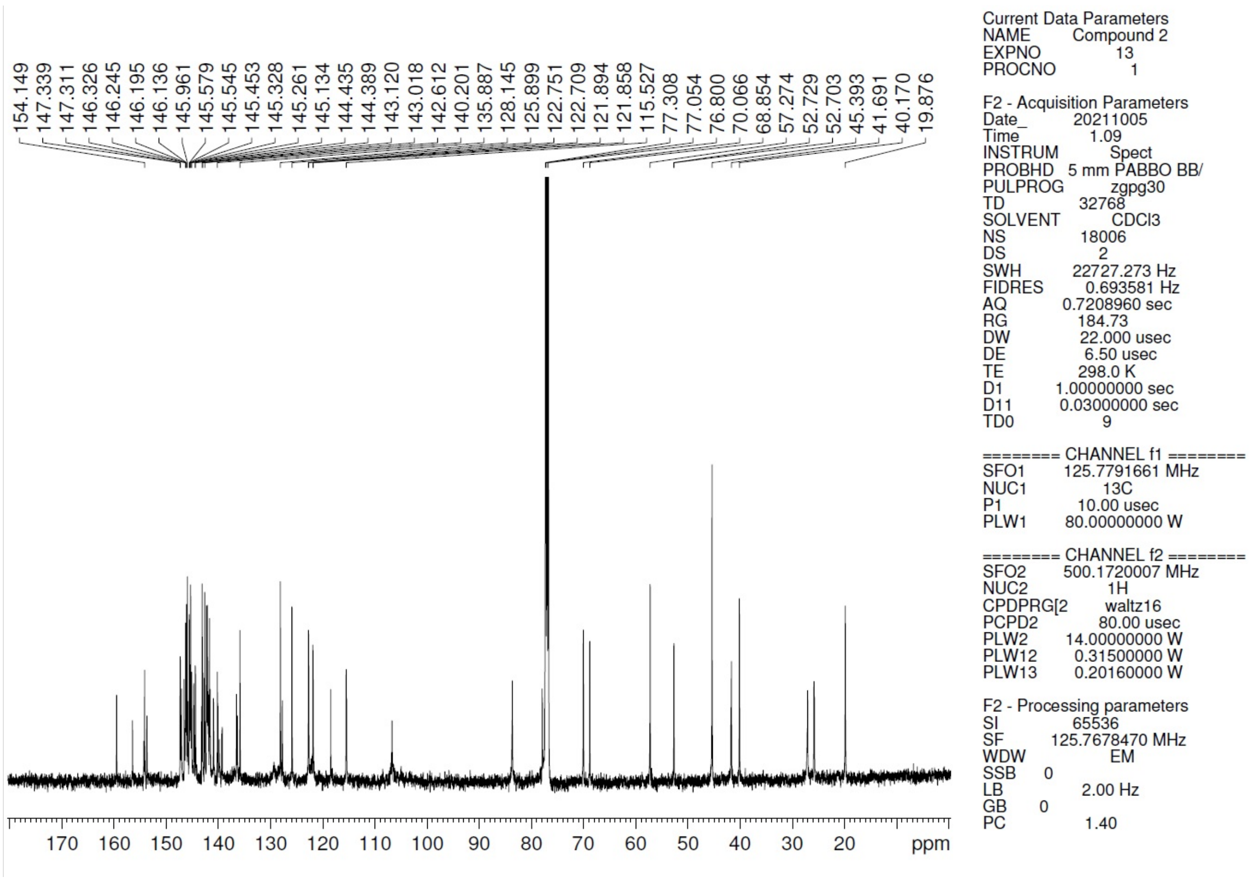

Figure S5. ^{13}C NMR spectra of compound **2** (125,78 MHz, $CDCl_3$).

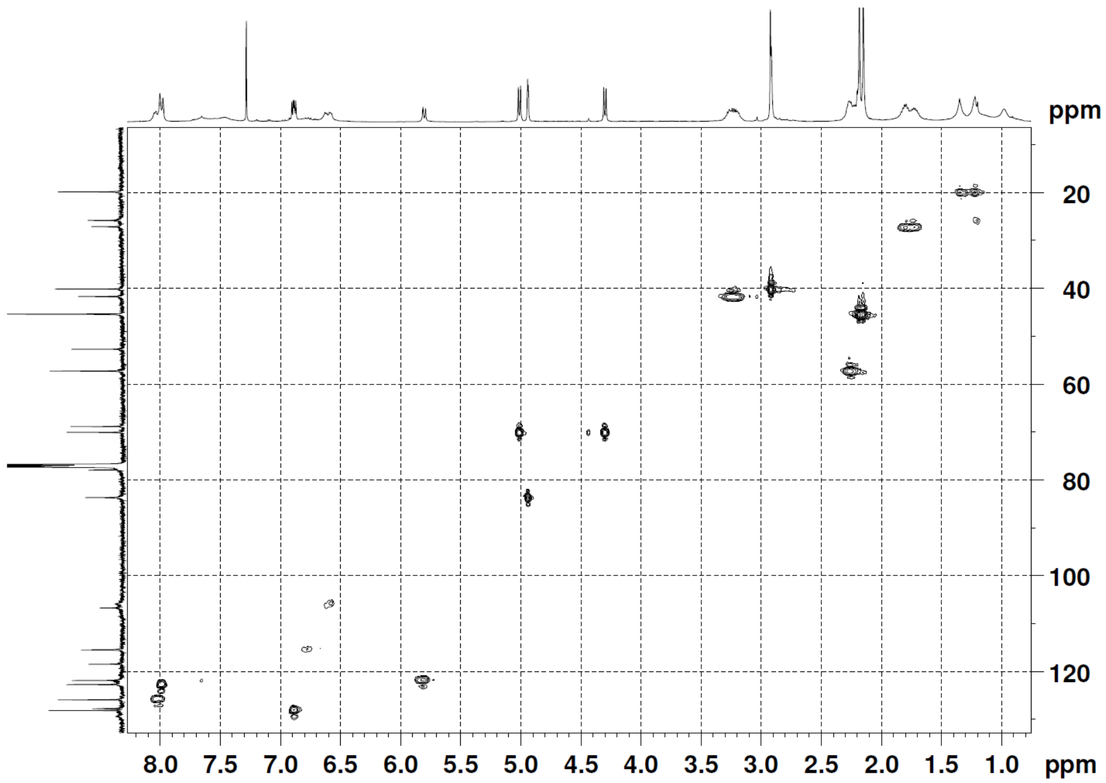

Figure S6. HSQC spectra of compound **2** (500.17 MHz for ^{1}H, 125.78 MHz for ^{13}C, $CDCl_3$).

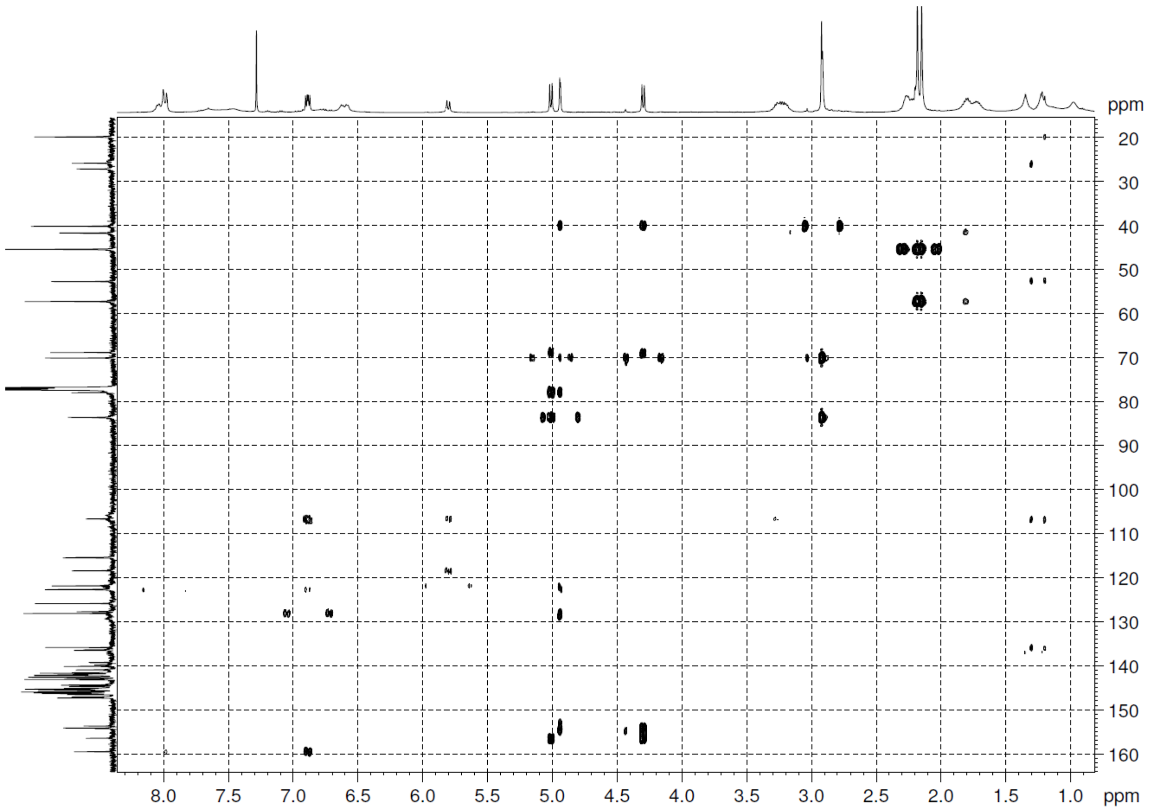

Figure S7. HMBC spectra of compound **2** (500.17 MHz for ^{1}H, 125.78 MHz for ^{13}C, $CDCl_3$).

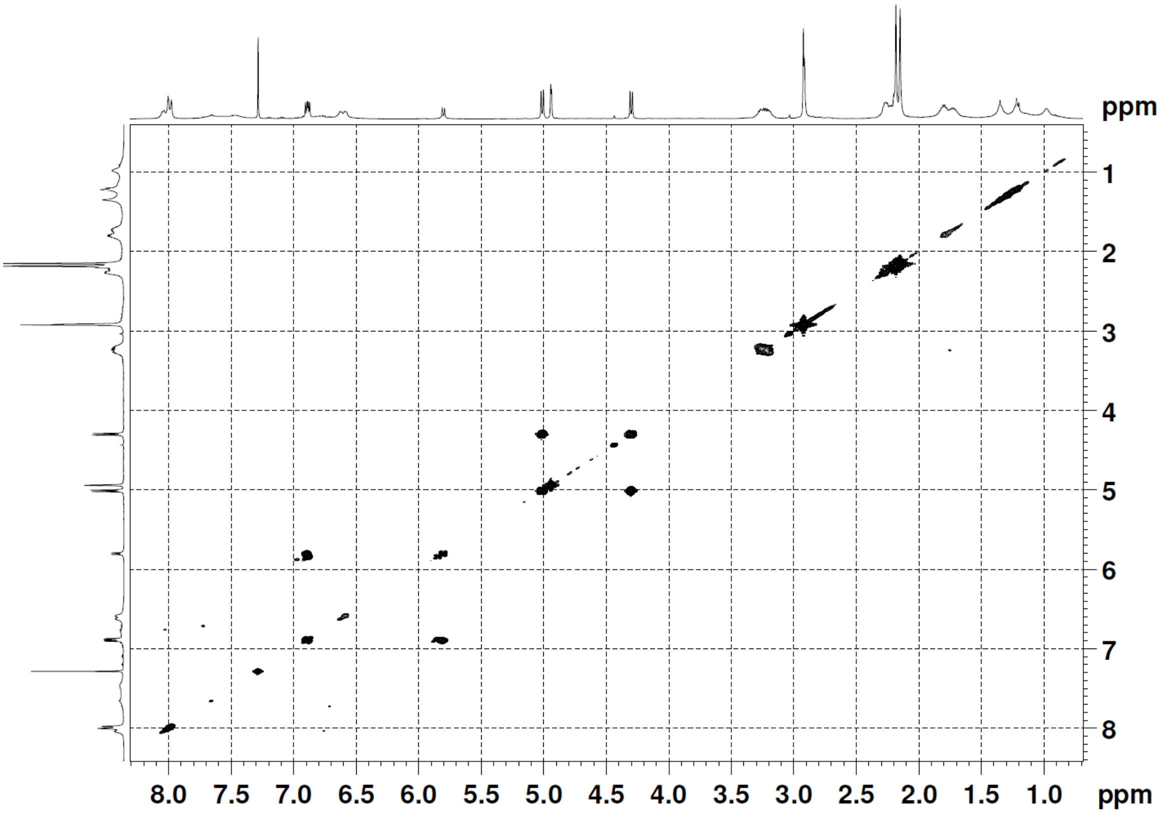

Figure S8. H-H COSY spectra of compound **2** (500.17 MHz for ^{1}H, $CDCl_3$).

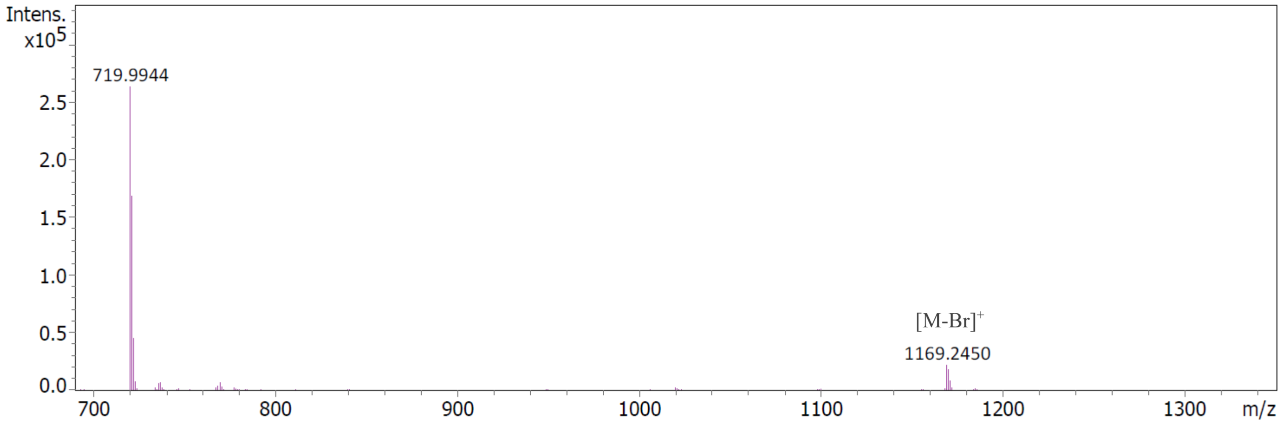

Figure S9. ESI-HRMS spectra of compound **2**.

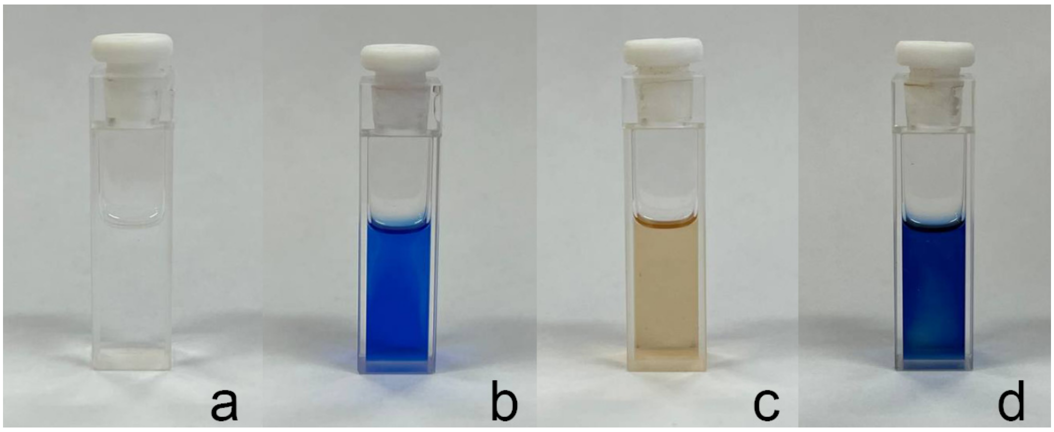

Figure S10. Photographs of a solutions **1** (a, b) and **2** (c, d) in THF (10^{-4} M) before (a, c) and after (b, d) UV-irradiation. T = 293 K.

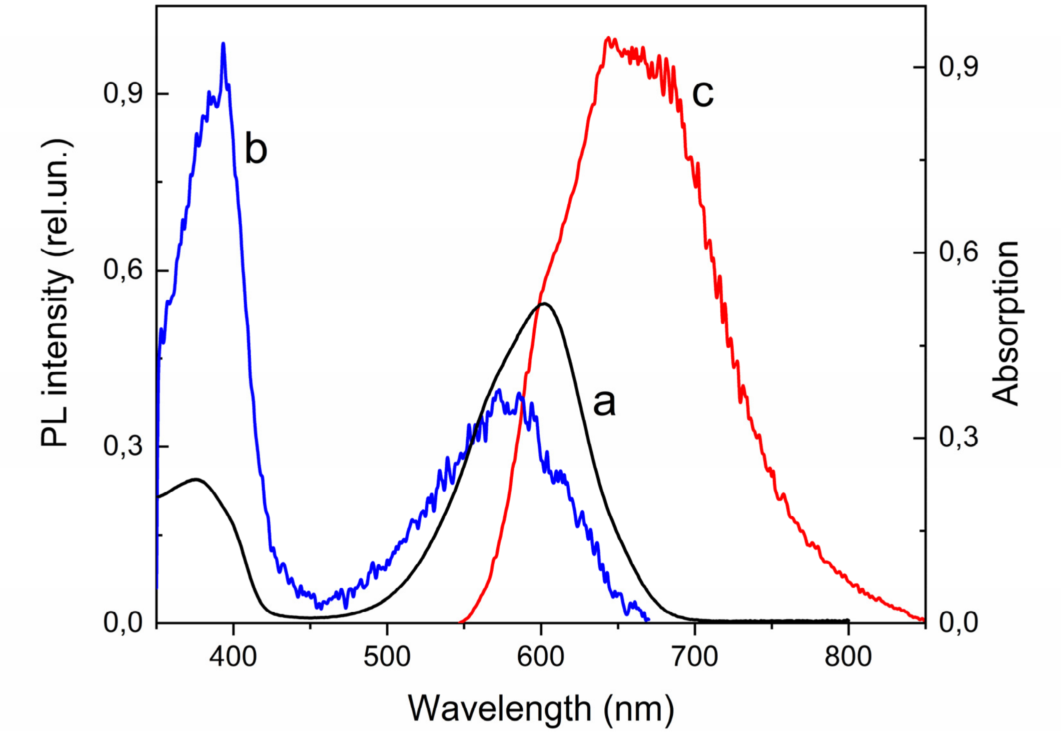

Figure S11. The absorption (a), PL excitation (b) and PL (c) spectra of **1`**. λ_{em} = 680 nm (2), λ_{exc} = 530 nm (3). T = 298 K.

References

1. Khuzin, A.A.; Galimov, D.I.; Tulyabaev, A.R.; Khuzina, L.L. Synthesis, Photochromic and Luminescent Properties of Ammonium Salts of Spiropyrans. *Molecules,* **2022**, 27, 8492–8498. https://doi.org/10.3390/molecules27238492
2. Nilsson, J.R.; Li, S.; Önfelt, B.; Andréasson, J. Light–induced cytotoxicity of a photochromic spiropyran. *Chem. Commun.*, **2011**, 47, 11020–11022. https://doi.org/10.1039/C1CC13561A
